# Extraction and Analysis of Natural Color From Beetroot ( *Beta vulgaris* L.) Using Different Techniques, and Its Utilization in Ice Cream Manufacturing

**DOI:** 10.1002/fsn3.70167

**Published:** 2025-04-18

**Authors:** Salman Khalil, Salah Laaraj, Nida Firdous, Umar Farooq, Mohamed Bouhrim, Rashed N. Herqash, Abdelaaty A. Shahat, Ashiq Hussain, Aziz Mouhaddach, Bruno Eto, Aliza Batool, Barira Bibi, Amina Ayesha, Farooq Arshad, Kaoutar Elfazazi

**Affiliations:** ^1^ Faculty of Food and Home Sciences Muhmmad Nawaz Shareef University of Agriculture Multan Pakistan; ^2^ Agri‐Food Technology and Quality Laboratory, Regional Centre of Agricultural Research of Tadla National Institute of Agricultural Research (INRA) Rabat Morocco; ^3^ Biological Engineering Laboratory, Faculty of Sciences and Techniques Sultan Moulay Slimane University Beni Mellal Morocco; ^4^ Laboratoires TBC, Laboratory of Pharmacology, Pharmacokinetics, and Clinical Pharmacy Faculty of Pharmaceutical and Biological Sciences Lille France; ^5^ Department of Pharmacognosy College of Pharmacy, King Saud University Riyadh Saudi Arabia; ^6^ Institute of Food Science and Nutrition, University of Sargodha Sargodha Pakistan; ^7^ Vegetable and Microbial Biotechnology, Biodiversity and Environment Faculty of Sciences, Mohammed V University in Rabat Rabat Morocco; ^8^ University Institute of Biochemistry and Biotechnology PMAS Arid Agriculture University Rawalpindi Rawalpindi Pakistan

**Keywords:** antioxidant activity, *Beta vulgaris* L, cold press extraction, ice cream, natural colorant, Soxhlet extraction, ultrasonic extraction

## Abstract

A rich source of natural color, beetroot (Beta vulgaris L. subsp. vulgaris) extracts and colorants offer deep red color to the dishes and boost their nutritional and antioxidant value. This research extracted beetroot color using Soxhlet, cold press, and ultrasonic methods. After testing the extracts for yield %, color (L*, a*, and b*), betalains, total polyphenols, and antioxidant activity, the colorant was used to make ice cream. The results of three techniques revealed that the extract obtained from Soxhlet extraction (SE) has more total phenolic content (244.11 mg GAE/100 g), DPPH free radical scavenging activity (23.41%), significantly higher yield (48.05%), and better color results, as compared to the other extraction techniques. However, the contents of betalains (399.47 mg/L) were observed more in the extract obtained from ultrasonic extraction (UE). The extract obtained after the SE technique was further utilized in the ice cream manufacturing to check its acceptability in this product, compared to the artificial color. Six samples of ice cream were developed having different ratios of beetroot‐derived color and artificial color. Control ice cream (T0) contained artificial color, whereas ice cream (T5) was developed by replacing complete artificial color with beetroot‐derived color. The other treatments (T1), (T2), (T3), and (T4) contained a combination of both natural and artificial colors with different proportions. Various quality parameters of these ice cream formulations, such as pH, acidity, brix, melting rate, overrun, and specific gravity, were studied. The result signified that the addition of beetroot color did not have a considerable effect on these attributes. The result of color analysis of ice cream suggested that the mean value of L* is higher in T5 (73.16), which only contains 0.1% beetroot color, and is lowest in T0 (65.24), which contains 0.1% artificial color. The addition of natural colorant resulted in a higher L* value. Sensory characteristics including color, mouth coating, flavor, aroma, texture, and overall acceptability of T5 showed more acceptance and significant results as compared to other treatments that contained artificial colors. Therefore, the beetroot‐derived natural colorants could be employed to develop nutritional, healthy, and acceptable ice cream.

## Graphical abstract

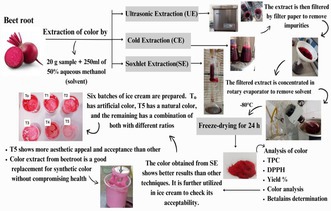

## 1 Introduction

Color is the most crucial sensory aspect of any food product that typically influences how well a food product performs on the market. Color additives could be utilized for a variety of operations such as the standardization of the colors of raw materials, providing color to the commodities that would be colorless otherwise, and also compensating for color losses under various processing conditions (Silva et al. 2022). The primary purpose of food coloring additives is to restore color lost as a result of the interaction of food with air, temperature, light, and various storage conditions (Sharma et al. 2023). Consumer perception and expectation of freshness and naturalness for food products involve the non‐existence of additives like artificial colors, sweeteners, preservatives, and flavors. At the same time, consumers believe food additives like natural colorants to be safe (Echegaray et al. 2023). Synthetic colors are a complex blend of metal ions, oxygen, hydrogen, carbon, nitrogen, and sulfur (Duan et al. 2023). Some naturally occurring food colors have been prepared synthetically to enhance their attributes for use in commercial applications. Color production has advanced significantly over the last few decades because naturally occurring pigments are unstable and have heat and pH sensitivity, so their usage is not that sustainable (Lan et al. 2023). Scientists have therefore created formulations that yield naturally similar colors to meet the pigment requirement and to make them stable as well. The benefits and drawbacks of using artificial colors in various food items have always been a topic of debate from the beginning. Focusing on the detrimental effects of artificial dyes on public health, customers are now more interested in safe substitutes such as naturally derived colors (Dufossé 2006; Carocho et al. 2015; Mota et al. 2023).

To improve the quality and organoleptic attributes of food products, many synthetic food colorants were developed and added. However, with time, the majority of these colorants were banned or became illegal because of their obvious adverse effects on consumer health, signs of short‐ and long‐term toxicity, and potential for health impairment including cancer risk (Amchova et al. 2024; Dewan et al. 2024). In addition to allergic reactions, several reports have revealed that synthetic food coloring has a big influence on how kids' behavior changes from positive to negative (Gostner et al. 2015; Masone and Chanforan 2015). In the food sector, natural additives such as bioactive compounds and natural colorants have gained significant importance in recent times. Enhanced food safety, improved nutritional content, and sensory attributes are a few benefits driving natural color trends in the food industry (Zin et al. 2020). Natural color pigments are organic and are nontoxic. In addition, higher concentrations of naturally occurring pigments are also permitted as compared to artificial food colorants (Gahlawat 2019). Natural pigments, out of which red is the most widely utilized color to captivate customers, have potential applications as food additives (Orlandi et al. 2022). Whereas red color is also extensively used in the manufacturing of ice cream; therefore, the extraction and utilization of red color from plant sources was selected for the current work.

The red beetroot ( *Beta vulgaris* L. subsp. *vulgaris*) belongs to the Amaranthaceae family. Beetroot is thought to have derived its name from the Greek letter beta because of the way the swollen root looks like a Greek B. This annual plant grows upright when it has tuberous rootstocks. It is considered one of the top ten vegetables in terms of antioxidant capacity (Carrillo et al. 2019; Bashir et al. 2024). Beetroot extracts are a rich source of natural pigments, polyphenols, and antioxidant compounds (Bashir et al. 2024). Not only the edible part of beetroot but also the waste streams of its processing have been found to be good sources of natural dyes (Lazăr et al. 2021; Sooch et al. 2024). There have been several techniques tested to extract and preserve the natural colors from the beetroots, such as Soxhlet extraction (SE), ultrasonic‐assisted extraction (UE) and cold extraction (CE) (Borjan et al. 2022). Additionally, several encapsulating, drying, and extraction methods have been developed to create betalains in dried forms from the beetroot for increased stability (Tutunchi et al. 2019). While the use of spray drying and ultrasound assistance in food processing and preservation has been proven to show technological advantages (Fatima et al. 2023; Hussain, Batool et al. 2024). There are uses for dried beetroot in cheese and edible coatings (Punia Bangar et al. 2023). Betalains, which are isolated from beetroots, are approved as food additives and are designated as E‐162. When E‐162 is added to any food, it turns its shade into orange‐red (Aykın‐Dinçer et al. 2021). Beetroot is utilized as a food coloring agent to add color to various dairy items such as ice cream, yogurts, sausages, candies, jellies, and chicken frankfurters. Furthermore, adding beetroot to food as an additive or dietary supplement may improve its nutritional content, bioactivities, and sensory attributes (Amnah 2013; Thiruvengadam et al. 2024).

In the modern era, consumer pleasure is linked not only to the flavor, smell, appearance, and attractive gaze of food items but also to their impact on health, improved quality of life, and lifespan (Hussain et al. 2023; Hussain, Kausar et al. 2024). Ice cream has evolved as one of the favorite desserts of all ages of people, which is not only tasty but also nutritious. However, the stability and safety of this product are always a concern for its producers (Mohammed et al. 2023). Further, ice cream is considered a poor source of phytochemicals and antioxidants (Goraya and Bajwa 2015). Ice cream is a frozen dairy product that has been emulsified. Air bubbles and milk fat globules are mixed with protein, sugar, water, and ice crystals. Many innovative ice cream development techniques have been launched as a result of growing demand and scientific advancements. These techniques include the use of natural plant‐based colorants and additives as well as the use of functional ingredients like plant powders and extracts that are high in bioactives (Das and Hooda 2023). Plant‐based food colors, such as those derived from beetroot have been demonstrated to be a promising substitute for synthetic food colors in the production of ice cream, given the growing demand for non‐toxic and environmentally friendly food coloring agents. Red beetroot was utilized to extract the natural color, which worked well in the ice cream (Ali and Jameel 2023); however, this research was limited to the formal analysis of the developed ice cream. It is generally believed that consuming synthetic food colorants can cause both allergic reactions and food intolerance, so consumers believe food additives like natural colorants to be safe. Therefore, there is a need to introduce such natural colorants that will satisfy customers' desire to enhance the quality of food as well as impart health benefits. Beetroot is an excellent replacement for artificial color due to its vibrant, natural hue and health benefits (Sooch et al. 2024). Rich in antioxidants and nutrients, beetroot not only adds a deep red color to foods but also enhances their nutritional profile (Punia Bangar et al. 2023). Unlike synthetic dyes, beetroot extracts and colorants pose no health risks (Thakur and Modi 2024). According to studies, betalains, which are found naturally in beets, give off a bright and consistent red color and are a healthier substitute for artificial dyes, especially Red Dye 40. In ice cream recipes, beetroot pigment has demonstrated the ability to endure storage, retaining its color and sensory attributes for prolonged periods of time without fading. Because it offers a natural remedy for the need for environmentally sustainable and health‐conscious food coloring, this is very important (Ali and Jameel 2023). It may enhance the perceived healthiness of the ice cream by incorporating plant‐derived colorants known for their nutritional benefits. It also meets the preferences of consumers seeking products with clean labels, free from artificial additives and synthetic colorants. Therefore, the objectives of this study were to extract natural food color from beetroot using different extraction techniques, to compare the influence of processing techniques on the yield of color, and then to assess the stability and applicability of beetroot (red beet) derived color in ice cream at various levels replacing the commercially used dye.

## 2 Materials and Methods

The plant materials used in this study are cultivated in the author's country (Pakistan), therefore no legal requirement was required for their cultivation and utilization.

### 2.1 Procurement of Raw Materials

Fresh beetroots ( *Beta vulgaris* L.), fully mature and having a uniform color and shape, were procured from a local market near MNS‐University of Agriculture Multan (MNSUA). All the chemicals and reagents were made available in the laboratory of the Department of Food Science and Technology and the central laboratory system of MNS‐University of Agriculture Multan. Raw materials for the development of ice cream were procured from a local market near the surrounding areas of MNS‐University of Agriculture Multan (MNSUA). All the chemicals and reagents were of analytical grade and were purchased from Sigma Aldrich, Germany.

### 2.2 Extraction of Color From Beetroot

Beetroot material was ground, and color was obtained by employing various extraction techniques such as SE, UE, and CE. All of the experiments were conducted using materials from a similar batch. The methodology as adopted by Borjan et al. (2022) was explored for multiple extraction techniques and aqueous methanol as solvent, such as 50% aqueous methanol, emphasizing its effectiveness in the extraction of betalain pigments. The betalain pigments from beetroot are efficiently dissolved in a 50% methanol and water mixture, producing a high color yield. The water component aids in expanding plant tissue, which facilitates the release of pigments, while methanol improves the extraction efficiency for polar chemicals like betalains. Afterward, the obtained extracts were evaporated by following the guidelines of Borjan et al. (2022), using a rotary evaporator and under reduced pressure, and the solvent was separated. The yield of extraction (mass of color or extract/mass of the dry material) was determined to represent the impact of the extraction techniques.

#### 2.2.1 Cold Extraction (CE)

CE of beetroot was carried out by following the guidelines given by Borjan et al. (2022). Beetroot was cut into uniform pieces, 1/8 to 1/4 in. thick in order to dry them. It was made sure the slices don't overlap and were arranged in a single layer on the electric dehydrator trays. After setting the dehydrator to 125°F–135°F (52°C–57°C), these slices were left to dry for 12 h, checking evenly to make sure drying. To improve surface area, dried beetroot was ground into a fine powder. Briefly explaining, a dry and powdered beetroot sample of 20 g and a solvent of about 250 mL was introduced into an Erlenmeyer flask, and the solvent used was 50% aqueous/methanol. Magnetic grain was further added to the system to avoid the continuous stirring, and it was then placed on a magnetic stirrer. The extraction from the sample took place for approximately 2 h at room temperature at about 150 rpm. The solution was then filtered using 0.15 cm Whatman filter paper.

#### 2.2.2 Soxhlet Extraction (SE)

Soxhlet apparatus was used to perform the SE, as was previously reported by Borjan et al. (2022). Briefly explaining, a 50% aqueous methanol was used as a solvent. Dried beetroot was ground into a fine powder, then a ground beetroot sample of 20 g was added to the tube, and about 250 mL of the same solvent as described in the earlier section was introduced into the flask. Solvent was heated, evaporated, and condensed, dripping onto beetroot powder. Color components were extracted by constant solvent circulation and gathered in a flask. Extraction from the sample was done for around 3 h in three cycles. The temperature was set according to the boiling point of the solvent, which was 60°C. The solution was then filtered using 0.15 cm Whatman filter paper.

#### 2.2.3 Ultrasonic Extraction (UE)

UE of the beetroot sample was performed after following the procedure given by Borjan et al. (2022). To improve the surface area, dried beetroot is ground into a fine powder. A dry and ground beetroot sample of 20 g and a solvent of about 250 mL were introduced into an Erlenmeyer flask, and 50% aqueous methanol was used as a solvent. Then at a fixed power of 40 kHz, the Erlenmeyer flask was placed into the ultrasonic bath. The level of liquid in the Erlenmeyer flask was kept lower than the level of the bath. For around 2 h at a constant temperature of 40°C, the extraction was performed. The solution was then filtered using 0.15 cm Whatman filter paper.

### 2.3 Freeze Drying of Extracted Color

Freeze drying of the extracted color was performed according to the guidelines of Aykın‐Dinçer et al. (2021), with required modifications. The extract of beetroot was concentrated in a rotary evaporator at 40°C temperature and 200‐mmHg of vacuum pressure. For further analysis, some quantities of the concentrated extracts were adjusted to 25° brix and the remaining extracts were freeze‐dried. After spreading the concentrated extract over trays in a thin layer, it was frozen at −80°C for almost 24 h. The trays were then placed into a freeze dryer after the process of freezing. At a temperature of −40°C (shelf temperature) and an absolute chamber pressure of 5333 Pa (40 mmHg), the freeze‐drying process was initiated. It took almost 48 h for the process to reach room temperature. The resulting color in powder form was stored in an amber‐colored glass jar that was properly hermetically sealed until it was further examined and used in product development.

### 2.4 Analyses of Color Extracted From Beetroot

Color analyses were performed before its utilization in ice cream to check the yield and quality of the extracted color. The main goal of these analyses was to check the quality of the extracted color.

#### 2.4.1 Total Phenolic Content Determination

The total phenolic content of the color was analyzed according to the procedure explained by Hussain et al. (2021). Briefly explaining, 1 mL of sample was added into 10 mL of the extraction mixture (Methanol: 90 mL, Acetone: 8 mL, and HCL: 2 mL). The mixture was then homogenized and poured into falcon tubes. The tubes were then introduced into a vortex for a few min. After removing from the vortex, the tubes were then placed into a centrifuge at elevated temperature for 4–5 Min. Then 400 μL of supernatant from centrifuged falcon tube and 800 μL of Folin–Ciocalteu Reagent were taken in separate falcon tubes and set into a vortex unit. Then 1600 μL of NaCO3 (700 Mm) was added into tubes were removed from the vortex machine and 200 μL of the sample was taken in a cuvette, and placed on a spectrophotometer (UV/VIS, Model 1201, Shimadzu, Kyoto, Japan). One blank sample was run in the same spectrophotometer for the reading. Absorbance was checked at 765 Nm.

#### 2.4.2 Antioxidant Activity Assay

The calculation of free radical scavenging activity of color was analyzed spectrophotometrically by the process given by Hussain et al. (2021). Briefly explaining, 1.5 mL of sample and 15 mL of extraction mixture were added and then subjected to homogenization. Then the homogenized sample was put into the Falcon tubes, which were subjected to the vortex for a few min. Then the tubes were removed from the vortex unit and were placed in a centrifuge at elevated temperature for 3–5 Min. After that, 150 μL of supernatant was taken from the centrifuged falcon tubes and poured into another falcon tube. DPPH solution was mixed in a concentration of about 15 mL with the classified sample. dpph solution was made by mixing 4 Mg of DPPH in 100 mL of methanol. Then these tubes were placed in darkness for about 30 min; after that, a small amount of sample was added to the Cuvette. the blank sample was placed in another cuvette on a spectrophotometer and absorbance was checked at 517 Nm. The total percentage of the radical scavenging activity was determined by using the following equation

$$\text{Radical Scavenging}\;\left(\%\right)=\left(1-A\;f/A\;0\right)\times 100$$

Where, A f = final absorbance and A 0 = initial absorbance.

#### 2.4.3 Beet Root Color Analysis

The color of the sample was analyzed according to the procedure explained by Sagdic et al. (2012), using the colorimeter (Lovibond Tintometer, Lovibond, Germany). With its visual parameter, it is specially designed to enhance the use of Lovibond's glass channels. A Lovibond colorimeter employs the principle of light reflection to detect color, and the color is then expressed in Lovibond units. Briefly explaining, a sample of 15 g was added to the beaker, and the instrument was run. Then the sample was adjusted onto the glass of the colorimeter, and light was allowed to pass from the surface sample. In L*, a*, and b* forms, readings are shown on the screen display, where the L* value determines the level of black to white (0–100), a* from red to green (+ for red and − for green) and b* from yellow to blue (+ for yellow and − for blue).

#### 2.4.4 Betalains Determination

The amount of betalains in the color was determined by following the method provided by Singh et al. (2017). First, 0.5 mL of beetroot extract was diluted into 50 mL of deionized water, and then spectrophotometrically at 480 and 538 nm, the ratio of betacyanins and betaxanthins in the extracts was analyzed. The absorbance reading obtained was used to calculate the betalain concentration for each sample. The content of betalain was then calculated using the equation as given below;

$$\text{Total Betalains}\;\left[\mathrm{mg}/L\right]=A\times \mathrm{DF}\times \mathrm{Mw}\times 1000/\varepsilon \times L$$

Whereas, A = Absorption, DF = The dilution factor, and L = Path length of the cuvette (1 cm).

For the quantification of betacyanins and betaxanthins, the molecular weights (Mw) and molar extinction coefficients (e) for the quantification of betacyanin are (MW = 550 g/mol, *e* = 60,000 L/mol cm in H_2_O) and betaxanthins are (MW = 308 g/mol, *e* = 48,000 L/mol cm in H_2_O) were employed.

#### 2.4.5 Gravimetric Analysis

The weight of the extract obtained was also calculated by following the process given by Sivakumar et al. (2009). To calculate the total amount of colorant or extract obtained from beetroot samples, after the UE, SE, and CE techniques, the samples were collected and filtered by filter paper and placed in dry, clean, and weighed glass dishes. The extracts were then dried in a freeze‐dryer till all solvent and water evaporated. The dried sample was then ground and weighed. To obtain a consistent weight, the steps of drying, cooling, and weighing were repeated, and the extract's weight was calculated. The yield for the total colorant or extract was then calculated using the following formula;

$$\begin{array}{c} \text{Total extract}/\text{colorant yield}\;\left(\%\right)=\\ \frac{\text{Total extract}/\text{colorant obtained}\;\left(g\right)\;}{\text{Amount of beetroot used}\;\left(g\right)}\times 100 \end{array}$$

### 2.5 Preparation of Ice Cream

#### 2.5.1 Experimental Design

The ice cream was prepared according to the process given by Sutar et al. (2010), with slight modifications. Six batches of ice cream were produced by different ratios of beetroot‐derived color or extract and artificial color, as shown in Table 1. Control ice cream was made without using beetroot‐derived color or extract. The color extract from beetroot (obtained through SE) was utilized in different proportions with artificial color in comparison to a control group, which only contains artificial color. Similarly, another batch of ice cream was developed with the complete replacement of the artificial color with a beetroot‐derived color or extract. Table 1 shows the experimental plan of ice cream preparation.

**TABLE 1:** Experimental design for planned research.

Treatment	Beetroot extract (%)	Artificial color (%)
T_0_	—	0.1
T_1_	0.04	0.06
T_2_	0.05	0.05
T_3_	0.06	0.04
T_4_	0.07	0.03
T_5_	0.1	—

#### 2.5.2 Preparation of Ice Cream

**FIGURE 1:**
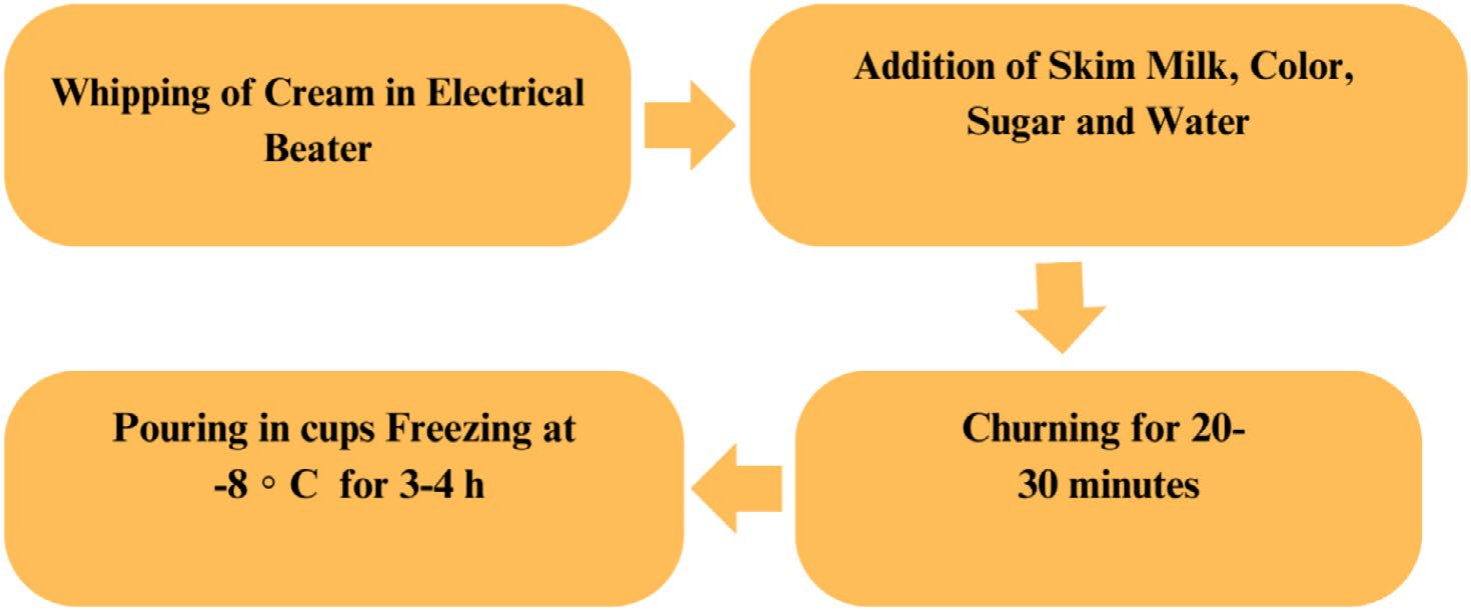
Flow diagram of ice cream preparation.

For the preparation of ice cream, 45% of whipping cream was taken, and in an electric beater, cream whipping was done. Then, in a separate bowl, 10% of sugar, 30% of milk powder, and whipped cream were mixed. Then, all the ingredients were mixed by using an electrical beater properly. At the same time, in this mixture, beetroot‐derived color and 15% of water were added. Water helps to dissolve sugar and other ingredients, ensuring even distribution throughout and also improving ice cream texture. After churning for 20–30 min, when the desired consistency and texture were achieved, the ice cream obtained was first packed and then placed in the freezer at −18°C for 3 to 4 h. Figure 1 presents the flow line of ice cream preparation.

#### 2.5.3 Ice Cream Color Analysis

The color of different formulations of ice cream (melted sample) was analyzed according to the same procedure explained by Sagdic et al. (2012), as explained earlier for the color determination of beetroot extract.

#### 2.5.4 Ice Cream Brix Determination

A refractometer was used to determine the ice cream sample's brix at 25°C ± 2°C, according to the procedure mentioned by Siddique et al. (2024). Additionally, the refractometer indicates the direct reading on the screen, indicating the number of total soluble solids in the sample under study. Briefly explain, the ice cream was placed at room temperature until it melted down; 2–3 drops of a sample of ice cream were inserted into the prism of the refractometer, and the upper plastic lid or cover was closed. Then, against a bright light source, the reading as brix degree was noted.

#### 2.5.5 Overrun of Ice Cream

The total volume of the air integrated into ice cream is referred to as the overrun of the ice cream. With the addition of air, the overrun becomes 100%, and it doubles the total volume of the ice cream. Overrun is the maximum quantity of air that can be added to commercial ice cream. By taking guidelines from the study of Homayouni and Norouzi (2016) and employing the below‐given formula, the overrun of ice cream formulations was determined.

$$\begin{array}{c} \text{Overrun}\;\left(\%\right)=\\ \frac{\text{weight}.\;\text{of}\;a\;\mathrm{cup}\;\text{of the}\;\mathrm{mix}-\text{weight}.\;\text{of}\;a\;\mathrm{cup}\;\text{of}\;\mathrm{ice}\;\text{cream}}{\text{weight}.\;\text{of}\;a\;\mathrm{cup}\;\text{of}\;\mathrm{ice}\;\text{cream}}\times 100 \end{array}$$

#### 2.5.6 Specific Gravity of Ice Cream

Specific gravity is the ratio of the mass of a substance to the mass of a reference substance for a similar given volume. The specific gravity of the ice cream was analyzed by a procedure adopted by El‐Samahy et al. (2009) at 20°C. The unit of specific gravity was g/cm^3^. First of all, the total volume of the empty cup of ice cream was calculated by putting water in it. Then the total volume of the same cup was calculated by putting ice cream in it, and the below given formula was used to calculate the specific gravity.

$$\begin{array}{c} \text{Specific gravity}=\\ \frac{\text{Weight of}\;\mathrm{cup}\;\text{with}\;\mathrm{ice}\;\text{cream}\left(g\right)-\text{Weight of empty}\;\mathrm{cup}\left(g\right)}{\text{Weight of}\;\mathrm{cup}\left({\mathrm{cm}}^{3}\right)} \end{array}$$

#### 2.5.7 Ice Cream Viscosity Determination

An instrument that is used to analyze the viscosity of a substance is called a viscometer. A viscometer is used for fluids with viscosities that vary depending on the conditions of flow. The viscosity of the ice cream samples was measured by following the protocols provided by Siddique et al. (2024). First of all, 300 mL of ice cream sample was taken at 4°C in a beaker before freezing, to measure the viscosity; then, at the speed of 60 rpm, spindle number 2 of the viscometer was employed to take torque measurements. The spindle of the viscometer was dipped into the beaker, making sure to avoid the side and bottom walls touching the beaker. Then the reading was calculated on the screen presented in mPa units.

#### 2.5.8 Ice Cream Melting Rate Determination

Ice cream sample melting rates were estimated at room temperature (25°C ± 2°C) by following the process given by Karaca et al. (2009). Briefly 70 g of the ice cream sample was taken and put on a mesh suspended over a 100 mL cylinder at room temperature. Then the time was calculated when the first drop of the sample fell into the cylinder. Then the mL of sample melted for 45 min with a time interval of 5 min were noted.

#### 2.5.9 Ice Cream pH Determination

The pH is referred to as the negative log of hydrogen ion concentration. The pH of the ice cream sample was estimated by an instrument known as a pH meter according to the method given by Siddique et al. (2024). First of all, the pH meter was standardized and calibrated by using a buffer solution. Then, in a beaker, 20 g of ice cream sample was taken, and the electrode of the pH meter was immersed into the beaker with the sample. The reading was recorded on the display screen.

#### 2.5.10 Ice Cream Acidity Determination

The acidity of ice cream samples was estimated by following the procedure adopted by Fiol et al. (2017). Acidity represents the lactic acid percentage in the samples of ice cream. Equivalent weight of lactic acid = 90 g. First of all, a 10 g sample was taken in a 250 mL Erlenmeyer flask and then 90 mL of distilled water was added into it. Then two to three drops of phenolphthalein were also added to the flask. Then it was titrated against 0.1 N NaOH solution until a slight pink color appeared. The percentage of lactic acid was estimated by the following formula

$$\text{Lactic acid}\;\left(\%\right)=\text{Volume of}\;0.1\;N\;\text{NaOH used}\times 0.009$$

#### 2.5.11 Ice Cream Sensory Evaluation

Sensory evaluation of ice cream was done by taking guidelines from the study of Wichchukit and O'Mahony (2015) at the Muhammad Nawaz Shareef University of Agriculture, Multan, with the help of a semi‐trained panel of 45 members of both genders, having an average age of 40 years, to check the acceptability of ice cream. All the participants were informed about the evaluation process, and their informed consent was taken. The product was analyzed for its color, aroma, texture, flavor, taste, mouth coating, and overall acceptability on a 0–9 hedonic scale on which 0 was the lowest score and 9 the maximum score. The product was presented to the evaluators in the cups, which were coded, and performa were provided for scoring under standard laboratory conditions. Distilled water bottles were also provided for rinsing purposes.

### 2.6 Statistical Analysis

All the analyses were carried out in triplicate, and the results were reported as mean ± standard deviations. ANOVA (one‐way) was used for the statistical analysis. To compare the means at a significant level of *p* ≤ 0.05, Duncan's multiple range test was employed. The obtained data was statistically analyzed to determine the validity of the hypothesis using STATISTIX (Version 8.1) software (Montgomery, 2017).

## 3 Results and Discussion

### 3.1 Beetroot Color Extract

#### 3.1.1 Total Phenolic Content

The mean values of the TPC of SE, CE, and UE extracts of beetroot are shown in Table 2, from where significantly different results can be seen for the three techniques. The results of TPC analysis indicate that the extract obtained from SE contains a higher level of TPC, which is 244.11 ± 5.64 mg GAE/100 g, followed by the UE (188.38 ± 2.17 mg GAE/100 g), while the CE contains the least amount of TPC, which is 160.40 ± 1.60 mg GAE/100 g. This high efficiency of SE in extracting TPC from the beetroot extract sample, while requiring a relatively small amount of solvent and achieving a complete extraction, was possibly due to the continuous reflux process (Borjan et al. 2022). According to Bashir et al. (2024), the high concentration of bioactive phenolic components, such as phenolic acids and flavonoids, in beetroot extracts makes them a well‐known food source with significant nutritional and therapeutic benefits. Beetroot waste parts are also a good source of phenolic compounds (Sooch et al. 2024). The current research shows agreement with the findings of Borjan et al. (2022) who have studied the effect of extraction techniques on the beetroot extract. They observed that the extract obtained from SE has a TPC value ranging from 221 to 196 mg GAE/100 g. According to this study, the extract obtained from UE has a TPC value of 222–95 mg GAE/100 g, while concerning their study, the CE has a TPC value of 182–76 mg GAE/100 g. According to Otegbayo et al. (2020), beetroot beverages are a good source of TPC, as they observed TPC values ranging from 2587.48 to 4286.17 mg/L GAE in fermented beetroot beverage, and these higher values were possibly due to the fermentation process. Similarly, Panghal et al. (2017) also found beetroot drinks to be a good source of TPC. The use of novel extraction techniques like ultrasonic‐assisted extraction also proved useful in extracting beetroot, when Tutunchi et al. (2019) provided good results for TPC in the beetroot extracts, just as in the current work UE, after SE, also provided good results for TPC, possibly due to the cavitation process initiated by the UE (Kaur and Ghoshal 2024).

**TABLE 2:** TPC value of beetroot extract.

Extraction technique	TPC (mg GAE/100 g)
Soxhlet extraction (SE)	244.11 ± 5.64^a^
Cold extraction (CE)	160.40 ± 1.60^c^
Ultrasonic extraction (UE)	188.38 ± 2.17^b^

*Note:* Values are means along with standard deviations (*n* = 3), whereas, different small alphabetical letters indicate that results are significant (*p* ≤ 0.05).

#### 3.1.2 Antioxidant Activity

The results of the antioxidant activity (DPPH assay) of beetroot extracts obtained from different techniques are shown in Table 3, showing significantly different values. The results of the DPPH assay indicate that the UE and SE have a higher level of antioxidant activity with values of 26.87% ± 1.33% and 23.41% ± 2.64%, respectively. While the extract obtained from CE has a significantly low antioxidant value of 15.29% ± 4.57%. These results could also be related to the results of the TPC given in Table 2, as the TPC of the beetroot extracts may have contributed to the DPPH activity. Betalains have also been found to show antioxidant properties both in vivo and in vitro conditions (Martínez‐Rodríguez et al. 2022). According to a recent study, rice fortified with betalains can be used as a functional grain to enhance health when taken as a raw dietary supplement (Fu et al. 2020). Beetroot extracts are an excellent natural source of antioxidant compounds (Bashir et al. 2024; Sooch et al. 2024). In addition to having a high phenolic acid content, red beets have large antioxidant potential and can also be utilized to produce a natural color (Antigo et al. 2017). The current research shows agreement with the findings of Borjan et al. (2022), as they also studied the effect of extraction techniques on beetroot extract. According to their studies, UE with 50% methanol (MeOH) has more DPPH free radical scavenging activity than SE and CE with 50% methanol (MeOH). According to the results of Tutunchi et al. (2019), the beetroot extracts possess high antioxidant activity if green extraction techniques like ultrasound‐assisted extraction are used, while they observed 59.87% ± 4.94% DPPH free radical scavenging activity of ultrasonic‐assisted extracts. The use of novel extraction techniques such as ultrasonic‐assisted extraction was also experimented with by Hussain, Arif et al. (2024), which helped to retain maximum antioxidants in the plant extracts, possibly due to the cavitation process that ruptures the plant cell walls, thus releasing antioxidant compounds.

**TABLE 3:** DPPH activity of beetroot extract.

Extraction technique	DPPH activity (%)
Soxhlet extraction (SE)	23.41 ± 2.64^b^
Cold extraction (CE)	15.29 ± 4.57^c^
Ultrasonic extraction (UE)	26.87 ± 1.33^a^

*Note:* Values are means along with standard deviations (*n* = 3), whereas, different small alphabetical letters indicate that results are significant (*p* ≤ 0.05).

#### 3.1.3 Color Values

The color values of beetroot extracts obtained from three different techniques are measured by the colorimeter, and the results are given in Table 4. According to the results, the color extract from SE has the lowest L* value, which is 26.60, which represents that it has a darker color as compared to the L* value of CE, which is 27.43, and UE, which is 28.21. Furthermore, the a* value measures the level of red to green; the positive value shows red while the negative value shows green. The result suggests that the color obtained from SE has a higher a* value, which is 0.56, followed by CE and UE at 0.09 and 0.06. The b* value measures the level of yellow and blue; the positive value shows yellow while the negative value shows blue color. The result suggests that UE has the lowest b* value, 0.58, as compared to CE and SE, which are 0.86 and 0.8. The current study results are in line with the findings of Otegbayo et al. (2020), who studied the physicochemical properties of beetroot wine produced at varying fermentation days. From their results, the value of L* ranged from 29.16 to 39.04. Another research was conducted by Dervisoglu et al. (2005), who studied the effect of soy protein concentrate on the physical, chemical, and sensory characteristics of ice cream, and the a* value was found to be from −1.94 to 7.03. A similar trend was found for the values of b* in a study conducted by Singo and Beswa (2019). They studied the effect of roselle extract on selected quality parameters of ice cream. The value of b* ranged from 7.13 to 0.43, respectively. These variations in the color values of the beetroot extracts were possibly due to the differences in the extraction of polyphenols, carotenoids, and other coloring compounds, which may have differed due to the varying parameters of extraction protocols (Nirmal et al. 2021).

**TABLE 4:** Color values of beetroot extract.

Extraction technique	L*	a*	b*
Soxhlet extraction (SE)	26.60 ± 0.47 ^c^	0.56 ± 0.03^a^	0.80 ± 0.03^b^
Cold extraction (CE)	27.43 ± 0.54 ^b^	0.09 ± 0.05^b^	0.86 ± 0.32^a^
Ultrasonic extraction (UE)	28.21 ± 0.05 ^a^	0.06 ± 0.08^c^	0.58 ± 0.25^c^

*Note:* Values are means along with standard deviations (*n* = 3), whereas, different small alphabetical letters indicate that results are significant (*p* ≤ 0.05).

#### 3.1.4 Betalain Content

The total betalains content of beetroot extract obtained from SE, CE, and UE is represented in Table 5. The result indicates that the extract obtained from UE has the highest value of betalains content, 399.47 ± 3.38 mg/L, followed by the SE, 388.40 ± 10.79 mg/L. The amount of betalains is the least in the extract obtained by CE, 379.94 ± 6.42 mg/L. The result indicates that the extract obtained from UE has significantly higher TBc (betacyanin) and TBx (betaxhantins) values of 247.19 ± 2.00 mg/L and 152.28 ± 1.50 mg/L, respectively. Total betalains are the sum of both betaxanthins and betacyanins. In addition to being utilized in functional foods and drink compositions, betalains have been used as natural colorants in a variety of food items (Panghal et al. 2017). The current study results are in line with the findings of Singh et al. (2017), who studied the optimization of extraction of betalain pigments from *Beta vulgaris* peels by microwave pretreatment. The amount of betalains obtained by them with citric acid as solvent was in the range of 156.76–242.41 mg/L, while with ethanol the betalains value ranged from 459.24 to 234.44 mg/L. In the current study, methanol and water in equal ratio were used as a solvent, and the value of betalains obtained was found parallel to the values obtained by using ethanol. While methanol increases the extraction efficiency for polar compounds like betalains, the water component helps to stretch plant tissue, which enables the release of pigments (Borjan et al. 2022). Present findings were also supported by the results of Tutunchi et al. (2019), as they observed high extraction and stability of betalains in the ultrasound‐assisted extracts of the beetroot. Similarly, in the current work, higher contents of betalains in UE extracts might be due to the high extraction efficiency of this technique, which is known due to the cavitation process caused by the ultrasound waves (Righi Pessoa da Silva et al. 2018). In contrast to other traditional techniques, UE may have contributed to the cavitation‐induced disintegration of plant tissues and cell walls from the beetroot components, releasing additional betalain contents (Kaur and Ghoshal 2024). The primary pigments that give beetroot its color are betalains, which are found in this vegetable in large quantities (Sooch et al. 2024). According to Bashir et al. (2024), beetroot is the richest source of betalains, which could be extracted through the application of different techniques. Present values of betalains (betaxanthin and betacyanin) were significantly higher than those given by Zin et al. (2020), as they used conventional extraction techniques to draw colorants from the beetroot. Khan et al. (2015) infused fruit spread and juice of banana with betalains that were isolated from berries to create a natural color. The results indicated that after 6 months of storage at 5°C, fruit covered with betalains exhibited 40% stability. The application of naturally occurring antioxidants such as betalains is seen in many food items, as Attia et al. (2013) showed red beetroots have an impact on maize oil while assessing the oil's antioxidant capability. They reported 380 mg betalain/100 g on fresh weight of red beetroot. These applications of betalains in food products could be related to the use of beetroot color in the development of ice cream in the present study.

**TABLE 5:** Betalains contents of beetroot extracts.

Extraction technique	TBc (mg/L)	TBx (mg/L)	TB (mg/L)
Soxhlet extraction (SE)	239.75 ± 12.13^b^	148.65 ± 1.49 ^c^	388.40 ± 10.79^b^
Cold extraction (CE)	229.47 ± 5.56^c^	150.47 ± 1.16 ^b^	379.94 ± 6.42^c^
Ultrasonic extraction (UE)	247.19 ± 2.00^a^	152.28 ± 1.50 ^a^	399.47 ± 3.38^a^

*Note:* Values are means along with standard deviations (*n* = 3), whereas, different small alphabetical letters indicate that results are significant (*p* ≤ 0.05). Where, TBc = Total betacyanins (mg/L) TBx = Total betaxanthins (mg/L) TB = Total betalains contents (mg/L).

#### 3.1.5 Gravimetric Analysis

The results of the yield of extracts of beetroot obtained from the three given techniques are represented in Table 6. The result signifies that the maximum yield is obtained by SE, which is 48.05% ± 1.94%, followed by cold and UE, 42.30% ± 2.37% and 41.07% ± 2.26%, respectively. According to these results, the best treatment for the extraction of color concerning yield is represented by the sequence of SE > CE > UE. Present values of yield of extracts were comparatively higher than those provided by Zin et al. (2020) because they used conventional extraction procedures to extract colorant from the beetroot. The current research shows agreement with the results provided by Borjan et al. (2022), who studied the effect of different extraction techniques on the beetroot extract. Two conventional (SE and CE) and non‐conventional (UE) approaches were employed in their study while keeping in mind the fundamentals of green chemistry. The largest extraction yield was obtained from the water extraction particularly for the SE and UE, whereas the yield obtained from the CE was relatively lower as compared to previous techniques. The total extraction yields by using various solvents are represented in the following sequence: water > ethanol 50% > methanol 50% > methanol 30%. However, according to current research, the total extraction yield with a similar solvent is represented in the following sequence: SE with 50% methanol > CE with 50% methanol > UE with 50% methanol. The use of UE for beetroot color was also found effective when Sivakumar et al. (2009) found a good yield of the extracts obtained from this technology. SE for extracting color from beetroot is a better option due to its superior performance in comparison to CE and UE. Empirical results indicate that SE yields a higher quantity of extract, enhanced total phenolic content, greater antioxidant activity, and increased betalain content (Tables 2, 3, and 5). The continuous solvent circulation and prolonged extraction period inherent in the Soxhlet method ensure exhaustive extraction of beetroot's colorants (Sooch et al. 2024). This method's ability to maintain consistent temperature and solvent flow is crucial for efficiently extracting and preserving sensitive compounds. Supported by extensive research, SE is recognized for producing high‐purity extracts with minimal solvent usage, making it the optimal choice for achieving a potent and high‐quality beetroot color extract (Lazăr et al. 2021). Hence, in the current study, color extracted by SE was chosen for further application in ice cream.

**TABLE 6:** Yield (%) of beetroot color extract.

Extraction technique	Yield (%)
Soxhlet extraction (SE)	48.05 ± 1.94^a^
Cold extraction (CE)	42.30 ± 2.37^b^
Ultrasonic extraction (UE)	41.07 ± 2.26^c^

*Note:* Values are means along with standard deviations (*n* = 3), whereas, different small alphabetical letters indicate that results are significant (*p* ≤ 0.05).

### 3.2 Analysis of Ice Cream

#### 3.2.1 Color Analysis

**FIGURE 2:**
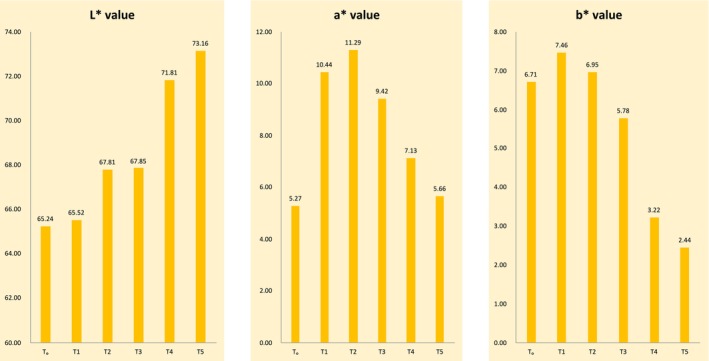
Color values (L*, a* and b*) of ice cream.

The color values of the ice cream for L*, a*, and b* are provided in Figure 2. According to the results of L* shown in Figure 2, T_5_, having natural colors obtained from beetroot at about 0.1%, shows the highest value of 73.16, followed by T_4_ at 71.81, which contains the combination of both natural colors of 0.07% and artificial color of 0.03%. T_0_, containing only artificial color in concentration of 0.1% and has the lowest L* value of 65.24. The mean value of a*, as shown in Figure 2, indicates that T_2_, which contains 0.05% of beetroot extract color and 0.05% of artificial color, has the highest a* value (11.29) followed by T_1_ (10.44), which contains 0.04% of beetroot color and 0.06% of artificial color. The result shows the lowest a* value in T_0_ (5.27), which only has 0.1% of artificial color. The mean value of b*, as also shown in Figure 2, indicates that T_1_, which contains 0.04% beetroot color and 0.06% artificial color, has the highest b* value of 7.46, followed by T_2_ (6.95) which contains 0.05% beetroot extract and 0.05% of artificial color. T_5_, which contains only beetroot color, has the lowest value of 2.44. The results were highly significant (*p* ≤ 0.01) for L*, a*, and b* values, as shown in Tables 7, 8 and 9. The first factor or parameter that guides consumers in the selection of food products is color. Various scientists explain that you choose food with your eyes first and then use your tongue to taste that food. The mean value of L* is higher in T_5_ at 73.16, which only contains 0.1% beetroot color, and is lowest in T_0_ at 65.24, which contains 0.1% artificial color. The increment of natural colorant concentration results in a higher L* value. As the concentration of beetroot color in ice cream decreases, the L* value decreases from T_5_ to T_0_. This is due to the reason that beetroot‐derived colorants provide pleasant light color to the ice cream, while the artificial color provides a darkish color. The results of the current study are similar to the findings of Singo and Beswa (2019), as they studied the effect of roselle extracts on the selected quality characteristics of ice cream. Various samples were analyzed for different parameters, and among all those, color was also one parameter. The L* value of ice cream from their results was found to range from 80.30 to 65.07. Another similar research was performed by Panwar and Kapoor (2019), as they studied the survival of probiotics in low‐fat ice creams enriched with prebiotic β‐manno‐oligosaccharides under simulated gastrointestinal stress. According to their study, L* value ranges from 81.59 to 78.06. The mean value of a*, as shown in Figure 2, indicates that the ice cream containing the combination of both artificial color and beetroot color has more redness than those that only contain artificial color and beetroot color, and thus have lower a* values. Similar research was also conducted by Dervisoglu et al. (2005), with inline findings as in that study. T_0_ has the lowest a* value (−1.94) and this color was turned into red with increasing the amount of flavor and color. The a * value, according to their findings, was found ranging from −1.94 to 7.03. A similar trend for the b* value was also found from the values of b* in a study conducted by Singo and Beswa (2019), as they found that the value of b* ranged from 7.13 to 0.43, respectively. Results from a similar study conducted by Roriz et al. (2018) also showed a similar pattern for color values of the ice cream, which was developed using artificial betalains, colorants from the beetroot, and from an algal species. In contrast to T_0_, which contains artificial and purified colorant, T_5_'s lower values of a* and b* and higher value of L* may be explained by the fact that this ice cream formulation contains natural beetroot colorant, which may present some opacity because these compounds were not purified (Roriz et al. 2018; Putradamni and Pramitasari 2024).

**TABLE 7:** ANOVA for L* value of ice cream.

Source	DF	SS	MS	*F*	*P*
Treatment	5	159.300	31.8600	133.54**	0.0000
Error	12	2.863	0.2386		
Total	17	162.163			

*Note:* **, Highly Significant; NS, non‐significant.

**TABLE 8:** ANOVA for a* value of ice cream.

Source	DF	SS	MS	*F*	*P*
Treatment	5	96.725	19.3450	64.96**	0.0000
Error	12	3.573	0.2978		
Total	17	100.298			

*Note:* **, Highly Significant; NS, non‐significant.

**TABLE 9:** ANOVA for b* value of ice cream.

Source	DF	SS	MS	*F*	*P*
Treatment	5	66.0982	13.2196	166.01**	0.0000
Error	12	0.9556	0.0796		
Total	17	67.0538			

*Note:* **, Highly Significant; NS, non‐significant.

#### 3.2.2 Brix Analysis

Brix is defined as the sugar content in any food matrix or solution. In the formation of ice cream, only the color concentration has been changed, whereas the sugar concentration throughout the system remained the same. The analysis of variance results exhibited that treatments' influence on samples is non‐significant (*p* ≥ 0.05). This suggests that changes in color concentration do not have a significant effect on the Brix of ice cream. The value of Brix was found to be ranging from 37.00 to 37.07 in different ice cream formulations developed in this work. The results indicate that changes in color concentration do not have a significant effect on the Brix of ice cream until the complete color is replaced with beetroot extract. The current study results follow the findings of Alizadeh et al. (2014), as they have studied the impact of Stevia on the physicochemical, sensory, rheology, and glycemic index of soft ice cream and observed a slight increase in the brix as a result of the addition of Stevia extracts. Different samples were analyzed, and the results of Brix were found ranging from 32.0 to 35.0. Present findings were also in line with the findings of Gisulga (2018), who focused on the physicochemical and sensory evaluation of probiotic malunggay ice cream. Their study highlighted that the value of Brix remained the same across different treatments. However, an opposite trend of brix was seen when Singo and Beswa (2019) used higher levels of roselle extracts (up to 15%) in the development of ice cream. The reason behind this increase in Brix of ice cream formulations having artificial color totally replaced with natural extracts is that these extracts could contribute some sugars to the ice cream formulations (Putradamni and Pramitasari 2024).

#### 3.2.3 Overrun

**FIGURE 3:**
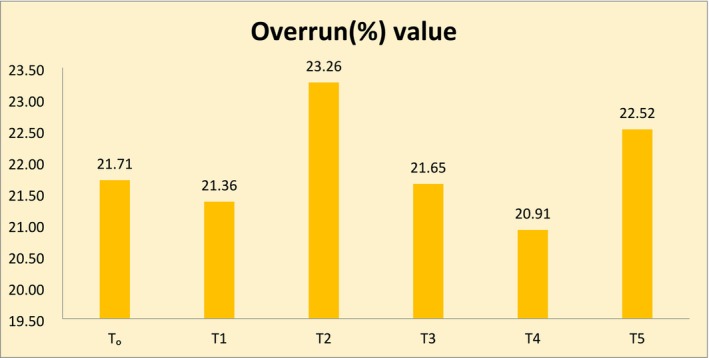
Overrun (%) of ice cream.

Overrun is referred to as the amount of air incorporated in ice cream during its processing (Putradamni and Pramitasari 2024). The results of overrun of ice cream are shown in Figure 3, having the mean values of different treatments of the ice cream. The result indicates that T_2_, which contains 0.04% of beetroot color and 0.06% of artificial color, has a higher overrun value of 23.26%, followed by T_5_ (22.52%), which contains 0.1% of beetroot color. T_4_, which has 0.07% of beetroot color and 0.03% of artificial color, has the lowest mean value of 20.91. The current research results are in agreement with the findings of Singo and Beswa (2019), as they have studied the effect of roselle extracts on the selected quality characteristics of ice cream. Overrun of different ice cream samples they observed was in the range between 35.5% to 17.6%. They observed a high overrun for the ice cream as a result of the addition of low levels of roselle extracts. The overrun of the ice cream was also found to be affected similarly when Mohammed et al. (2023) used red beetroot extracts‐based emulsions to develop ice cream, which was compared to the control. Similar significantly different results for the overrun of ice cream were also observed by Sutar et al. (2010) when they developed ice cream using soy extracts from seven different varieties. The high overrun value of T5 and T2 might be explained in this way: beetroot‐derived colorants might have increased the total solids of the ice cream formulations, which in turn resulted in higher overrun values, as the natural extracts' higher concentration of non‐water ingredients, such as sugar, permits more air to be added during the freezing process, which results in the creation of air pockets that enhance the amount of ice cream with the same weight of mix (Hacıbektaşoğlu and Gündoğdu 2024; Putradamni and Pramitasari 2024).

#### 3.2.4 Specific Gravity

The mean values of the specific gravity of ice cream were found to be ranging from 0.72 to 0.73 g/cm^3^. The result indicates that changes in color concentration do not have a significant effect on the specific gravity of the ice cream. Overall, the specific gravity of ice cream samples is almost the same, with a minute variation, which possibly was due to negligible fat contents in the beetroot extracts. The current research results regarding specific gravity are in line with the findings of El‐Samahy et al. (2009), as they studied the characteristics of ice cream incorporated with the concentrated pulp of cactus pear. The mean values of specific gravity of different samples of ice cream they observed were found ranging between 0.71 to 0.86 g/cm^3^. One another similar research by Goraya and Bajwa (2015) also supported the current results, as they studied the nutritional and functional attributes of ice cream processed with gooseberry. Their results of specific gravity ranged between 0.532 to 0.602 g/cm^3^. Sutar et al. (2010) observed a slight decrease in the specific gravity of the ice cream when they used soy extracts to develop ice cream, which might be due to a difference in the chemical composition of soy and beetroot extracts.

#### 3.2.5 Viscosity

The analysis of variance results exhibited that treatments' influence on the viscosity of the ice cream samples was non‐significant (*p* ≥ 0.05). This suggests that changes in color concentration do not have a significant effect on the viscosity of the ice cream. The mean values of the viscosity for the ice cream ranged from 126.28 to 126.31 mPa. Overall, the viscosity of ice cream samples was remained almost the same. Present findings are in line with the results of the study performed by Singo and Beswa (2019), who found a slight decrease in the viscosity of the ice cream, in which artificial color was replaced with different levels of the roselle extracts. The viscosity of the ice cream was reduced as its volume grew, yet its resistance to melting increased. In addition to giving ice cream a light texture, air also affects its physical characteristics, including viscosity and melting rate. The current research results are also in line with the findings of de la Cruz Martínez et al. (2020), who estimated the viscosity of ice cream mixture during batch crystallization in a scraped surface heat exchanger. The viscosity ranged from 50 to 150 mPa. The viscosity of ice cream was also found to be having same trend is in current work, when Sutar et al. (2010), used soy extracts from different varieties to develop ice cream formulations. As viscosity of ice cream is affected by variations in the fat, protein and other solid contents, and beetroot extracts did not contribute much of these solids, that's why the viscosity was found unaffected in different formulations (Hacıbektaşoğlu and Gündoğdu 2024; Putradamni and Pramitasari 2024).

#### 3.2.6 Melting Rate

The mean values of the melting rate of different ice creams were found to be ranging from 1.92 to 1.97 mL/min. The result indicates that the change in color concentration does not have a significant impact on the melting rate of the ice cream. Our research findings were found near to the values provided by Pon et al. (2015), as their results of the melting rate of ice cream were in the range of 1.50–1.09 mL/min. The melting rate of ice cream was significantly affected in a dissimilar pattern when Sutar et al. (2010) used soy extracts in the development of ice cream, which possibly was due to the different fat contents of soy extracts than beetroot extracts. The melting rate of ice cream was found to be increasing from 0.74 to 2.33 g/min when Singo and Beswa (2019) used roselle extracts at a higher level than our study in the manufacturing of ice cream. Similar findings were also found when Dervisoglu et al. (2005) added soy protein concentrate at various levels to develop ice cream. The network of fat globules generated during freezing, the size distribution of the air cells, and the poor stability of the air cells are typically the causes of the high melting rate of ice cream samples with high overrun. The primary microstructural elements of ice cream are ice, fat globules, and air. These constituents greatly impact the melt‐through or drip‐through characteristics of ice cream (Sofjan and Hartel 2004). There was no significant difference in the viscosity of these ice cream formulations since the ratios of air, ice crystals, and fat, as well as the storage temperature, all have an impact on the melting rate of ice cream. These parameters were nearly the same for all formulations.

#### 3.2.7 pH

The results of the pH of ice cream having different combinations of artificial and beetroot‐derived colorants ranged between 6.70–6.71. According to these results, the change in color concentration in the ice cream does not have a significant effect on the value of pH. There is a very slight difference in the pH between different treatments, as the overall pH of all treatments somehow remained unaffected by changing the color concentration. The current study results are in line with the findings of Markowska et al. (2023), when they studied the physicochemical properties and melting behavior of ice cream fortified with multimineral preparation from red algae. According to them, the pH of ice cream was found to be around 6.62. Another similar research work by Ranadheera et al. (2013) studied the physicochemical changes in the probiotic‐enriched ice cream prepared from the milk of a goat. From their findings, the pH range of different ice cream samples was between 6.61 to 6.65. The stability of betalains over a wide pH range throughout the heat treatment process gives them special advantages as a natural organic colorant (Fu et al. 2020), while in present findings, the pH showed non‐significant variations, which was a positive aspect for these beetroot‐derived natural colorants. A similar behavior in the pH of ice cream was also observed by Ali and Jameel (2023), when artificial dye in the ice cream was replaced by beetroot‐derived natural colorant.

#### 3.2.8 Acidity

The results of the acidity of ice cream having different combinations of artificial and beetroot‐derived colorants ranged from 0.21% to 0.23%. The overall results indicate that the change in color concentration in the ice cream does not bring a significant change in the acidity of the ice cream. The acidity of ice cream remained unaffected by changing the color concentrations; however, replacing the complete color with beetroot extract slightly affected the acidity. The current research shows agreement with the results of Markowska et al. (2023), as they studied the physicochemical properties and melting behavior of ice cream fortified with multimineral preparation from red algae. They found minimal variation in acidity among ice cream samples with different stabilizers and emulsifiers. Overall, the acidity also remained consistent in their findings. Further, similar results were also observed when Singo and Beswa (2019) added roselle extracts in the manufacturing process of ice cream and found a similar non‐significant increase in the acidity of the ice cream. They stated that the high concentration of organic acids in the extracts might have increased the acidity of ice cream samples, just as in the current work the acidity of the ice cream formulation having completely replaced artificial color with beetroot‐derived colorant was 0.23%, as compared to control developed with 100% artificial color and having 0.21%.

#### 3.2.9 Sensory Evaluation

**FIGURE 4:**
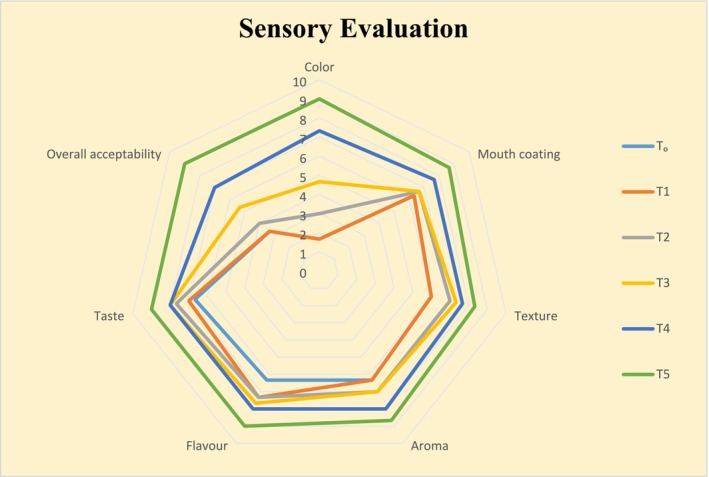
Sensory parameters of ice cream.

**FIGURE 5:**
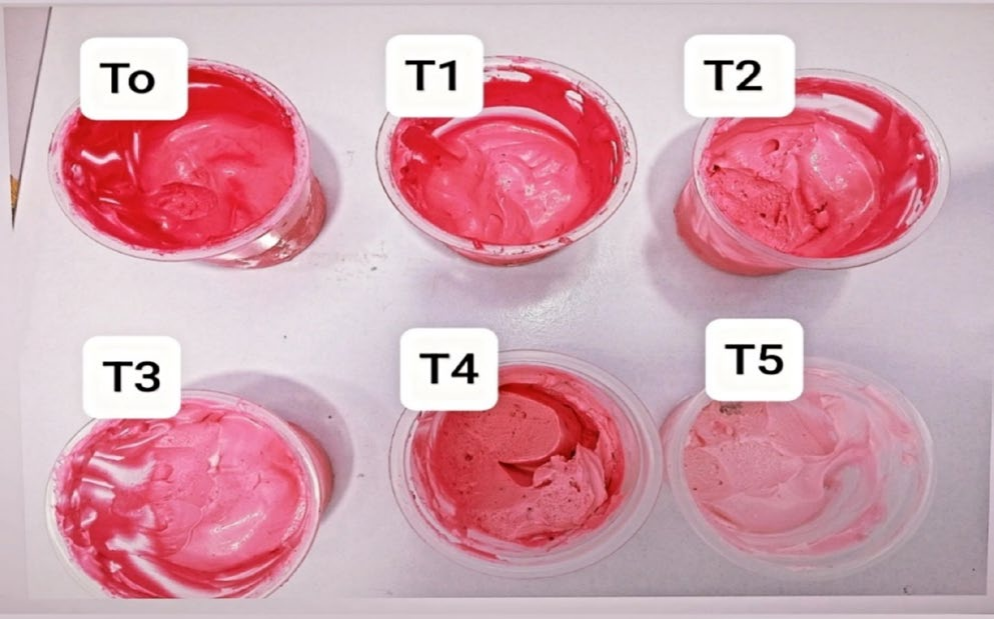
Treatments of ice cream.

Ice cream produced by using different ratios of color including beetroot‐derived color and artificial color was observed to have varying sensory scores. Various sensory properties such as color, mouth coating, texture, aroma, taste, flavor, and overall acceptability were included in the analyses. The mean values of all these parameters are shown in Figure 4. As the concentration of beetroot‐derived color increased and artificial color decreased from T_0_–T_5_, the score of overall acceptability was found to be increased. The higher value of overall acceptability was observed in T_5_ (9.00) with 0.1% of beetroot color. Images of different treatments of ice cream are also shown in Figure 5. The results of color, mouth coating, aroma, flavor, overall acceptability, and taste were highly significant (*p* **≤** 0.01) as shown in Tables 10, 11, 12, 13, 14, 15, 16. Current research findings were found to be near the observations of Goraya and Bajwa (2015), as they studied the nutritional and functional qualities of gooseberry mixed ice cream. The results of sensory scores from their study for the overall acceptability of gooseberry incorporated ice cream were in the range from 8.16 to 7.29. More recent findings of Mohammed et al. (2023) have shown that ice cream developed by incorporation of beetroot extracts did not show any negative aspects of sensory parameters, while the color of this ice cream was found stable during 6 months of storage. This possibly was due to the high stability of beetroot‐derived betalains. The findings of Sagdic et al. (2012) were also supportive of the current results of the sensory analysis when different extracts and essential oils were incorporated into the ice cream, which was equally liked by the consumers. The current findings were also validated by Aykın‐Dinçer et al. (2021) when they found that the use of beetroot extract and powder had a favorable impact on the sensory appearance, color, flavor, and overall approval of sausages. Attia et al. (2013) studied the utilization of red beet extract as a natural colorant in jelly and ice sherbets to enhance their sensory qualities and analyzed their acceptability, which was found to be based on the number of betalains they contain. Present findings for high acceptance of beetroot extract added ice cream were also supported when beetroot extracts were added to the ice creams as a natural color pigment, which improved the acceptability and stability of the product over 180 days at −20°C (Roriz et al. 2018). Color is thought to be related to flavor, which affects the perception of the quality of the food items and fascinates the cravings of consumers. Multiple naturally‐derived colors are utilized to enhance the physical appearance of the food products (Downham and Collins 2000). Betalains, which provide colors ranging from yellow to violet, are employed in a variety of dairy products, where consumers have given preferences to these products (Jurić et al. 2022). Several studies have shown that three factors such as betalain supply and profile, food matrix composition, and storage conditions are very important for incorporating betalains into food items as natural color, affecting the final acceptability of the product (Moussa‐Ayoub et al. 2016). The results of Attia et al. (2013) experiments using analysis of variance for the sensory evaluation of prepared jelly and ice sherbets showed that the highest scores for color, taste, and overall acceptability were given to jelly samples containing 0.30% and ice sherbets containing 0.20% red beetroot pigments, which is comparable to synthetic color carmine. Therefore, the high color scores for beetroot extract containing ice cream could be related to the role of these pigments provided through the incorporation of extracts.

**TABLE 10:** ANOVA table for color of ice cream.

Source	DF	SS	MS	*F*	*P*
Treatment	5	139.778	27.9556	38.71**	0.0000
Error	12	8.667	0.7222		
Total	17	148.444			

*Note:* **, Highly Significant; NS, non‐significant.

**TABLE 11:** ANOVA table for Mouth coating of ice cream.

Source	DF	SS	MS	*F*	*P*
Treatment	5	12.9444	2.58889	7.77**	0.0018
Error	12	4.0000	0.33333		
Total	17	16.9444			

*Note:* **, Highly Significant; NS, non‐significant.

**TABLE 12:** ANOVA table for Texture of ice cream.

Source	DF	SS	MS	F	*P*
Treatment	5	12.9444	2.58889	3.88^NS^	0.0252
Error	12	8.0000	0.66667		
Total	17	20.9444			

**TABLE 13:** ANOVA table for Aroma of ice cream.

Source	DF	SS	MS	*F*	*P*
Treatment	5	13.1111	2.62222	7.87**	0.0017
Error	12	4.0000	0.33333		
Total	17	17.1111			

*Note:* **, Highly Significant; NS, non‐significant.

**TABLE 14:** ANOVA table for Flavor of ice cream.

Source	DF	SS	MS	*F*	*P*
Treatment	5	11.6111	2.32222	10.45**	0.0005
Error	12	2.6667	0.22222		
Total	17	14.2778			

*Note:* **, Highly Significant; NS, non‐significant.

**TABLE 15:** ANOVA table for taste of ice cream.

Source	DF	SS	MS	*F*	*P*
Treatment	5	10.2778	2.05556	18.50**	0.0000
Error	12	1.3333	0.11111		
Total	17	11.6111			

*Note:* **, Highly Significant; NS, non‐significant.

**TABLE 16:** ANOVA table for overall acceptability of ice cream.

Source	DF	SS	MS	*F*	*P*
Treatment	5	78.0000	15.6000	23.40**	0.0000
Error	12	8.0000	0.6667		
Total	17	86.0000			

*Note:* **, Highly Significant; NS, non‐significant.

## 4 Conclusion

Beetroot is an excellent replacement for artificial color due to its vibrant, natural hue and health benefits. Rich in antioxidants and nutrients, beetroot not only adds a deep red color to foods but also enhances their nutritional profile. The current research was conducted to extract natural color from the beetroot, using three different techniques such as SE, CE, and UE. The influences of processing techniques were compared to get good quality results. After extracting color, various parameters were studied for each technique like TPC, antioxidant activity, color, betalains contents, and total yield %. A comparison of the extracts showed that the SE is the better option for color extraction, as these extracts exhibited a good percentage of yield, TPC, antioxidant value, and color results. Then this Soxhlet extracted color was further utilized in the ice cream to check its acceptability by comparing it with the artificial color. The overall results for color analysis were significant (*p* ≤ 0.01), but non‐significant (*p* ≥ 0.05) for brix, overrun, specific gravity, viscosity, melting rate, and overrun. The sensory evaluation of all the treatments of ice cream showed that the maximum score for overall acceptability was documented for ice cream which contained only beetroot color. Thus, beetroot color extracted through the Soxhlet technique produced good quality ice cream with optimum physicochemical parameters, which was highly preferred by the consumers. So, natural colorants and extracts from the beetroot could be derived using novel techniques, with the purpose of utilization in different food formulations.

## Author Contributions

**Salman Khalil:** investigation (equal), methodology (equal), resources (equal), writing – original draft (equal). **Salah Laaraj:** conceptualization (equal), methodology (equal), resources (equal), validation (equal), writing – original draft (equal), writing – review and editing (equal). **Nida Firdous:** formal analysis (equal), writing – review and editing (equal). **Umar Farooq:** data curation (equal), resources (equal), visualization (equal). **Mohamed Bouhrim:** methodology (equal), supervision (equal), visualization (equal), writing – review and editing (equal). **Rashed N. Herqash:** funding acquisition (equal), project administration (equal), writing – review and editing (equal). **Abdelaaty A. Shahat:** funding acquisition (equal), project administration (equal), writing – review and editing (equal). **Ashiq Hussain:** investigation (equal), methodology (equal), writing – original draft (equal), writing – review and editing (equal). **Aziz Mouhaddach:** resources (equal), writing – original draft (equal), writing – review and editing (equal). **Bruno Eto:** investigation (equal), visualization (equal), writing – review and editing (equal). **Aliza Batool:** investigation (equal), resources (equal), writing – review and editing (equal). **Barira Bibi:** data curation (equal), validation (equal), writing – review and editing (equal). **Amina Ayesha:** investigation (equal), resources (equal), writing – review and editing (equal). **Farooq Arshad:** data curation (equal), formal analysis (equal), resources (equal), software (equal). **Kaoutar Elfazazi:** supervision (equal), writing – original draft (equal), writing – review and editing (equal).

## Ethics Statement

There are no human or animal tests included in the present work study. However, for the sensory evaluation, the ethical committee of IFSN, UOS waived the requirement of ethical approval for the sensory analysis of the developed product. All the participants gave their written consent to participate in the analysis, and proper protocols were followed to safeguard their human rights.

## Conflicts of Interest

The authors declare no conflicts of interest.

## Data Availability

All the data relevant to this study have been provided within the manuscript.

## References

[fsn370167-bib-0001] Ali, R. T. M. , and Q. Y. Jameel . 2023. “Red Beetroot Betalains as a Novel Source of Colorant in Ice Cream as Compared With Red Dye 40 (E129).” Functional Foods in Health and Disease 13, no. 4: 225–239. 10.31989/ffhd.v13i4.1096.

[fsn370167-bib-0002] Alizadeh, M. , M. Azizi‐Lalabadi , and S. Kheirouri . 2014. “Impact of Using Stevia on Physicochemical, Sensory, Rheology, and Glycemic Index of Soft Ice Cream.” Food and Nutrition Sciences 5, no. 4: 390–398. 10.4236/fns.2014.54047.

[fsn370167-bib-0003] Amchova, P. , F. Siska , and J. Ruda‐Kucerova . 2024. “Food Safety and Health Concerns of Synthetic Food Colors: An Update.” Toxics 12, no. 7: 466. 10.3390/toxics12070466. 39058118 PMC11280921

[fsn370167-bib-0004] Amnah, M. 2013. “Nutritional, Sensory and Biological Study of Biscuits Fortified With Red Beet Roots.” Life Science Journal 10, no. 3: 1579–1584.

[fsn370167-bib-0005] Antigo, J. L. D. , R. D. C. Bergamasco , and G. S. Madrona . 2017. “Effect of pH on the Stability of Red Beet Extract (*Beta Vulgaris* L.) Microcapsules Produced by Spray Drying or Freeze Drying.” Food Science and Technology 38, no. 1: 72–77. 10.1590/1678-457X.34316.

[fsn370167-bib-0007] Attia, G. Y. , M. M. Moussa , and E. E. D. R. Sheashea . 2013. “Characterization of Red Pigments Extracted From Red Beet (*Beta Vulgaris*, L.) and Its Potential Uses as Antioxidant and Natural Food Colorants.” Egyptian Journal of Agricultural Research 91, no. 3: 1095–1110. 10.21608/ejar.2013.167086.

[fsn370167-bib-0009] Aykın‐Dinçer, E. , K. K. Güngör , E. Çağlar , and M. Erbaş . 2021. “The Use of Beetroot Extract and Extract Powder in Sausages as Natural Food Colorant.” International Journal of Food Engineering 17, no. 1: 75–82. 10.1515/ijfe-2019-0052.

[fsn370167-bib-0010] Bashir, R. , S. Tabassum , A. Adnan , A. Rashid , and A. Adnan . 2024. “Bioactive Profile, Pharmacological Attributes and Potential Application of *Beta Vulgaris* .” Journal of Food Measurement and Characterization 18: 3732–3743. 10.1007/s11694-024-02445-6.

[fsn370167-bib-0011] Borjan, D. , V. Šeregelj , D. C. Andrejč , et al. 2022. “Green Techniques for Preparation of Red Beetroot Extracts With Enhanced Biological Potential.” Antioxidants 11, no. 5: 805. 10.3390/antiox11050805. 35624669 PMC9138100

[fsn370167-bib-0012] Carocho, M. , P. Morales , and I. C. Ferreira . 2015. “Natural Food Additives: Quo Vadis?” Trends in Food Science & Technology 45, no. 2: 284–295. 10.1016/j.tifs.2015.06.007.

[fsn370167-bib-0013] Carrillo, C. , D. Wilches‐Pérez , E. Hallmann , R. Kazimierczak , and E. Rembiałkowska . 2019. “Organic Versus Conventional Beetroot. Bioactive Compounds and Antioxidant Properties.” Lebensmittel‐Wissenschaft & Technologie 116: 108552. 10.1016/j.lwt.2019.108552.

[fsn370167-bib-0014] Das, N. , and A. Hooda . 2023. “Chemistry and Different Aspects of Ice Cream.” In The Chemistry of Milk and Milk Products, 65–86. Apple Academic Press.

[fsn370167-bib-0015] de la Cruz Martínez, A. , R. E. Delgado Portales , J. D. Pérez Martínez , et al. 2020. “Estimation of Ice Cream Mixture Viscosity During Batch Crystallization in a Scraped Surface Heat Exchanger.” Processes 8, no. 2: 167. 10.3390/pr8020167.

[fsn370167-bib-0016] Dervisoglu, M. , F. Yazici , and O. Aydemir . 2005. “The Effect of Soy Protein Concentrate Addition on the Physical, Chemical, and Sensory Properties of Strawberry Flavored Ice Cream.” European Food Research and Technology 221: 466–470. 10.1007/s00217-005-1207-3.

[fsn370167-bib-1001] Dewan, M. F. , M. N. Islam , and M. S. Azam . 2024. “Food Additives/Preservatives and Their Implications for Human Health.” In Food Safety, 155–184. CRC Press. 10.1201/9781003333913.

[fsn370167-bib-0017] Downham, A. , and P. Collins . 2000. “Colouring Our Foods in the Last and Next Millennium.” International Journal of Food Science & Technology 35, no. 1: 5–22. 10.1046/j.1365-2621.2000.00373.x.

[fsn370167-bib-0018] Duan, L. , T. Zhou , Y. Zhang , et al. 2023. “Surface Optics and Color Effects of Liquid Metal Materials.” Advanced Materials 35, no. 26: 2210515. 10.1002/adma.202210515. 36709052

[fsn370167-bib-0019] Dufossé, L. 2006. “Microbial Production of Food Grade Pigments.” Food Technology and Biotechnology 44, no. 3: 313–321.

[fsn370167-bib-0020] Echegaray, N. , N. Guzel , M. Kumar , M. Guzel , A. Hassoun , and J. M. Lorenzo . 2023. “Recent Advancements in Natural Colorants and Their Application as Coloring in Food and in Intelligent Food Packaging.” Food Chemistry 404: 134453. 10.1016/j.foodchem.2022.134453. 36252374

[fsn370167-bib-0021] El‐Samahy, S. K. , K. M. Youssef , and T. E. Moussa‐Ayoub . 2009. “Producing Ice Cream With Concentrated Cactus Pear Pulp: A Preliminary Study.” Journal of the Professional Association for Cactus Development 11: 1–12.

[fsn370167-bib-0022] Fatima, P. , M. Nadeem , A. Hussain , et al. 2023. “Synergistic Effect of Microwave Heating and Thermosonication on the Physicochemical and Nutritional Quality of Muskmelon and Sugarcane Juice Blend.” Food Chemistry 425: 136489. 10.1016/j.foodchem.2023.136489. 37276674

[fsn370167-bib-0023] Fiol, C. , D. Prado , C. Romero , N. Laburu , M. Mora , and J. I. Alava . 2017. “Introduction of a New Family of Ice Creams.” International Journal of Gastronomy and Food Science 7: 5–10. 10.1016/j.ijgfs.2016.12.001.

[fsn370167-bib-0024] Fu, Y. , J. Shi , S. Y. Xie , T. Y. Zhang , O. P. Soladoye , and R. E. Aluko . 2020. “Red Beetroot Betalains: Perspectives on Extraction, Processing, and Potential Health Benefits.” Journal of Agricultural and Food Chemistry 68, no. 42: 11595–11611. 10.1021/acs.jafc.0c04241. 33040529

[fsn370167-bib-0025] Gahlawat, I. N. 2019. “Emerging New Insights Into Significance and Applications of Plant Pigments.” Journal of Integrated Science and Technology 7, no. 2: 29–34.

[fsn370167-bib-0026] Gisulga, J. B. 2018. “Production and Quality Evaluation of Probiotic Malunggay (*Moringa Oleifera* Lam.) ice Cream.” JPAIR Multidisciplinary Research 32, no. 1: 67–89. 10.7719/jpair.v32i1.576.

[fsn370167-bib-0027] Goraya, R. K. , and U. Bajwa . 2015. “Enhancing the Functional Properties and Nutritional Quality of Ice Cream With Processed Amla (Indian Gooseberry).” Journal of Food Science and Technology 52: 7861–7871. 10.1007/s13197-015-1877-1. 26604358 PMC4648887

[fsn370167-bib-0028] Gostner, J. M. , K. Becker , F. Ueberall , and D. Fuchs . 2015. “The Good and Bad of Antioxidant Foods: An Immunological Perspective.” Food and Chemical Toxicology 80: 72–79. 10.1016/j.fct.2015.02.012. 25698357

[fsn370167-bib-0029] Hacıbektaşoğlu, F. , and E. Gündoğdu . 2024. “Physicochemical, Nutritional, and Antioxidant Properties of Ice Cream Enriched With Red Beetroot (*Beta Vulgaris* L.) at Varying Sucrose Levels.” Turkish Journal of Agriculture‐Food Science and Technology 12, no. 10: 1722–1729. 10.24925/turjaf.v12i10.1722-1729.7053.

[fsn370167-bib-0030] Homayouni, A. , and S. Norouzi . 2016. “Evaluation of Physicochemical Traits, Sensory Properties and Survival of *Lactobacillus Casei* in Fermented Soy‐Based Ice Cream.” Journal of Food Processing and Preservation 40, no. 4: 681–687. 10.1111/jfpp.12648.

[fsn370167-bib-0031] Hussain, A. , M. R. Arif , A. Ahmed , et al. 2024. “Evaluation of Leaves, Flowers, and Seeds of Coriander ( *Coriandrum sativum* L.) Through Microwave Drying and Ultrasonic‐Assisted Extraction, for Biologically Active Components.” Journal of Food Processing and Preservation 2024, no. 1: 2378604. 10.1155/2024/2378604.

[fsn370167-bib-0032] Hussain, A. , A. Batool , S. Yaqub , et al. 2024. “Effects of Spray Drying and Ultrasonic Assisted Extraction on the Phytochemicals, Antioxidant and Antimicrobial Activities of Strawberry Fruit.” Food Chemistry Advances 5: 100755. 10.1016/j.focha.2024.100755.

[fsn370167-bib-0033] Hussain, A. , T. Kausar , A. Din , et al. 2021. “Determination of Total Phenolic, Flavonoid, Carotenoid, and Mineral Contents in Peel, Flesh, and Seeds of Pumpkin (*Cucurbita Maxima*).” Journal of Food Processing and Preservation 45, no. 6: e15542. 10.1111/jfpp.15542.

[fsn370167-bib-0034] Hussain, A. , T. Kausar , T. Siddique , et al. 2024. “Physiological and Biochemical Variations of Naturally Ripened Mango (*Mangifera Indica* L.) With Synthetic Calcium Carbide and Ethylene.” Scientific Reports 14, no. 1: 2121. 10.1038/s41598-024-52483-9. 38267498 PMC10808196

[fsn370167-bib-1002] Hussain, A. , S. Laaraj , T. Kausar , et al. 2023. “Through Incorporation in Wheat Flour to Boost Vitamin and Mineral Profiles of Formulated Biscuits.” International Journal of Food Science 2023, no. 1: 6654250. 10.1155/2023/6654250. 38025391 PMC10667046

[fsn370167-bib-0035] Jurić, S. , M. Jurić , Ż. Król‐Kilińska , et al. 2022. “Sources, Stability, Encapsulation and Application of Natural Pigments in Foods.” Food Reviews International 38, no. 8: 1735–1790. 10.1080/87559129.2020.1837862.

[fsn370167-bib-0036] Karaca, O. B. , M. Güven , K. Yasar , S. Kaya , and T. Kahyaoglu . 2009. “The Functional, Rheological and Sensory Characteristics of Ice Creams With Various Fat Replacers.” International Journal of Dairy Technology 62, no. 1: 93–99. 10.1111/j.1471-0307.2008.00456.x.

[fsn370167-bib-0038] Kaur, A. , and G. Ghoshal . 2024. “Comprehensive Analysis of Phytochemical Extraction of Betalains From *Beta Vulgaris* L. Pomace Using Conventional, Enzyme‐Assisted and Ultrasonic‐Assisted Methods.” Journal of Food Measurement and Characterization 19: 1–15. 10.1007/s11694-024-02997-7.

[fsn370167-bib-0039] Khan, M. I. , P. S. Harsha , A. S. Chauhan , S. V. N. Vijayendra , M. R. Asha , and P. Giridhar . 2015. “Betalains Rich *Rivina Humilis* L. Berry Extract as Natural Colorant in Product (Fruit Spread and RTS Beverage) Development.” Journal of Food Science and Technology 52: 1808–1813. 10.1007/s13197-013-1175-8. 25745261 PMC4348319

[fsn370167-bib-0040] Lan, T. , S. Qian , T. Song , H. Zhang , and J. Liu . 2023. “The Chromogenic Mechanism of Natural Pigments and the Methods and Techniques to Improve Their Stability: A Systematic Review.” Food Chemistry 407: 134875. 10.1016/j.foodchem.2022.134875. 36502728

[fsn370167-bib-0041] Lazăr, S. , O. E. Constantin , N. Stănciuc , I. Aprodu , C. Croitoru , and G. Râpeanu . 2021. “Optimization of Betalain Pigments Extraction Using Beetroot By‐Products as a Valuable Source.” Inventions 6, no. 3: 50. 10.3390/inventions6030050.

[fsn370167-bib-0042] Markowska, J. , A. Tyfa , A. Drabent , and A. Stępniak . 2023. “The Physicochemical Properties and Melting Behavior of Ice Cream Fortified With Multimineral Preparation From Red Algae.” Food 12, no. 24: 4481. 10.3390/foods12244481 PMC10742974 38137285

[fsn370167-bib-0043] Martínez‐Rodríguez, P. , M. A. Guerrero‐Rubio , P. Henarejos‐Escudero , F. García‐Carmona , and F. Gandía‐Herrero . 2022. “Health‐Promoting Potential of Betalains In Vivo and Their Relevance as Functional Ingredients: A Review.” Trends in Food Science & Technology 122: 66–82. 10.1016/j.tifs.2022.02.020.

[fsn370167-bib-0044] Masone, D. , and C. Chanforan . 2015. “Study on the Interaction of Artificial and Natural Food Colorants With Human Serum Albumin: A Computational Point of View.” Computational Biology and Chemistry 56: 152–158. 10.1016/j.compbiolchem.2015.04.006. 25935119

[fsn370167-bib-0045] Mohammed, A. N. , S. P. Ishwarya , and P. Nisha . 2023. “Ice‐Cream as a Model System to Evaluate the Food Colorant Functionality of Red Beet Extract Emulsion.” Journal of Culinary Science & Technology 21, no. 6: 960–980. 10.1080/15428052.2021.2024475.

[fsn370167-bib-0046] Mota, I. G. C. , R. A. M. D. Neves , S. S. D. C. Nascimento , B. L. L. Maciel , A. H. D. A. Morais , and T. S. Passos . 2023. “Artificial Dyes: Health Risks and the Need for Revision of International Regulations.” Food Reviews International 39, no. 3: 1578–1593. 10.1080/87559129.2021.1934694.

[fsn370167-bib-0047] Moussa‐Ayoub, T. E. , H. Jaeger , K. Youssef , et al. 2016. “Technological Characteristics and Selected Bioactive Compounds of *Opuntia dillenii* Cactus Fruit Juice Following the Impact of Pulsed Electric Field Pre‐Treatment.” Food Chemistry 210: 249–261. 10.1016/j.foodchem.2016.04.115. 27211645

[fsn370167-bib-0048] Nirmal, N. P. , R. Mereddy , and S. Maqsood . 2021. “Recent Developments in Emerging Technologies for Beetroot Pigment Extraction and Its Food Applications.” Food Chemistry 356: 129611. 10.1016/j.foodchem.2021.129611. 33838608

[fsn370167-bib-0049] Orlandi, V. T. , E. Martegani , C. Giaroni , A. Baj , and F. Bolognese . 2022. “Bacterial Pigments: A Colorful Palette Reservoir for Biotechnological Applications.” Biotechnology and Applied Biochemistry 69, no. 3: 981–1001. 10.1002/bab.2170. 33870552 PMC9544673

[fsn370167-bib-0050] Otegbayo, B. O. , I. M. Akwa , and A. R. Tanimola . 2020. “Physico‐Chemical Properties of Beetroot ( *Beta vulgaris* l.) Wine Produced at Varying Fermentation Days.” Scientific African 8: e00420. 10.1016/j.sciaf.2020.e00420.

[fsn370167-bib-0051] Panghal, A. , K. VirKAr , V. KumAr , S. B. Dhull , Y. Gat , and N. Chhikara . 2017. “Development of Probiotic Beetroot Drink.” Current Research in Nutrition and Food Science Journal 5, no. 3: 1–12. 10.12944/CRNFSJ.5.3.10.

[fsn370167-bib-0052] Panwar, D. , and M. Kapoor . 2019. “Enhanced Survival of Lactobacillus sp. in β‐Manno‐Oligosaccharides‐Enriched Low‐Fat Ice Cream Under Simulated Gastrointestinal Stress.” Journal of Food Processing and Preservation 43, no. 9: e14097. 10.1111/jfpp.14097.

[fsn370167-bib-0053] Pon, S. Y. , W. J. Lee , and G. H. Chong . 2015. “Textural and Rheological Properties of Stevia Ice Cream.” International Food Research Journal 22, no. 4: 1544–1549.

[fsn370167-bib-0054] Punia Bangar, S. , A. Singh , V. Chaudhary , N. Sharma , and J. M. Lorenzo . 2023. “Beetroot as a Novel Ingredient for Its Versatile Food Applications.” Critical Reviews in Food Science and Nutrition 63, no. 26: 8403–8427. 10.1080/10408398.2022.2055529. 35333666

[fsn370167-bib-0055] Putradamni, A. M. , and R. Pramitasari . 2024. “Formula Optimization, Physicochemical Characterization, and Sensory Evaluation of Beetroot‐Based Blended Frozen Dessert.” Food Chemistry Advances 4: 100672. 10.1016/j.focha.2024.100672.

[fsn370167-bib-0056] Ranadheera, C. S. , C. A. Evans , M. C. Adams , and S. K. Baines . 2013. “Production of Probiotic Ice Cream From Goat's Milk and Effect of Packaging Materials on Product Quality.” Small Ruminant Research 112, no. 1–3: 174–180. 10.1016/j.smallrumres.2012.12.020.

[fsn370167-bib-0057] Righi Pessoa da Silva, H. , C. Silva , and B. C. Bolanho . 2018. “Ultrasonic‐Assisted Extraction of Betalains From Red Beet (*Beta Vulgaris* L.).” Journal of Food Process Engineering 41, no. 6: e12833. 10.1111/jfpe.12833.

[fsn370167-bib-0058] Roriz, C. L. , J. C. Barreira , P. Morales , L. Barros , and I. C. Ferreira . 2018. “ *Gomphrena Globosa* L. as a Novel Source of Food‐Grade Betacyanins: Incorporation in Ice‐Cream and Comparison With Beet‐Root Extracts and Commercial Betalains.” Lebensmittel‐Wissenschaft & Technologie 92: 101–107. 10.1016/j.lwt.2018.02.009.

[fsn370167-bib-0059] Sagdic, O. , I. Ozturk , H. Cankurt , and F. Tornuk . 2012. “Interaction Between Some Phenolic Compounds and Probiotic Bacterium in Functional Ice Cream Production.” Food and Bioprocess Technology 5: 2964–2971. 10.1007/s11947-011-0611-x.

[fsn370167-bib-0060] Sharma, H. , G. Mahajan , M. Kaur , and R. Gupta . 2023. Microbes for Natural Food Additives, 169–203. Springer Nature Singapore. 10.1007/978-981-19-5711-6_8.

[fsn370167-bib-0061] Siddique, F. , A. Hussain , A. A. Mahdi , et al. 2024. “Comparison of Chemically Treated, Pasteurized, and Microwave‐Treated (At Different Time Durations) Chia Seeds Added to Mango‐Whey Beverage, During Different Storage Periods, for Physicochemical and Sensory Parameters.” Journal of Food Quality 2024, no. 1: 6688945. 10.1155/2024/6688945.

[fsn370167-bib-0062] Silva, M. M. , F. H. Reboredo , and F. C. Lidon . 2022. “Food Colour Additives: A Synoptical Overview on Their Chemical Properties, Applications in Food Products, and Health Side Effects.” Food 11, no. 3: 379. 10.3390/foods11030379. PMC8834239 35159529

[fsn370167-bib-0063] Singh, A. , M. Ganesapillai , and N. Gnanasundaram . 2017. “Optimizaton of Extraction of Betalain Pigments From *Beta Vulgaris* Peels by Microwave Pretreatment.” IOP Conference Series: Materials Science and Engineering 263: e032004.

[fsn370167-bib-0064] Singo, T. M. , and D. Beswa . 2019. “Effect of Roselle Extracts on the Selected Quality Characteristics of Ice Cream.” International Journal of Food Properties 22, no. 1: 42–53. 10.1080/10942912.2019.1567535.

[fsn370167-bib-0065] Sivakumar, V. , J. L. Anna , J. Vijayeeswarri , and G. Swaminathan . 2009. “Ultrasound Assisted Enhancement in Natural Dye Extraction From Beetroot for Industrial Applications and Natural Dyeing of Leather.” Ultrasonics Sonochemistry 16, no. 6: 782–789. 10.1016/j.ultsonch.2009.03.009. 19410496

[fsn370167-bib-0066] Sofjan, R. P. , and R. W. Hartel . 2004. “Effects of Overrun on Structural and Physical Characteristics of Ice Cream.” International Dairy Journal 14, no. 3: 255–262. 10.1016/j.idairyj.2003.08.005.

[fsn370167-bib-0067] Sooch, B. S. , N. Sandhu , M. K. Mann , and R. C. Ray . 2024. “Valorization of Beetroot Waste for Extraction of Natural Dye for Textile and Food Applications.” In Roots, Tubers, and Bulb Crop Wastes: Management by Biorefinery Approaches, 237–260. Springer Nature Singapore. https://link.springer.com/chapter/10.1007/978‐981‐99‐8266‐0_11.

[fsn370167-bib-0068] Sutar, N. , P. P. Sutar , and G. Singh . 2010. “Evaluation of Different Soybean Varieties for Manufacture of Soy Ice Cream.” International Journal of Dairy Technology 63, no. 1: 136–142. 10.1111/j.1471-0307.2009.00557.x.

[fsn370167-bib-0069] Thakur, M. , and V. K. Modi . 2024. “Biocolorants in Food: Sources, Extraction, Applications and Future Prospects.” Critical Reviews in Food Science and Nutrition 64, no. 14: 4674–4713. 10.1080/10408398.2022.2144997. 36503345

[fsn370167-bib-0070] Thiruvengadam, M. , I. M. Chung , R. Samynathan , et al. 2024. “A Comprehensive Review of Beetroot (*Beta Vulgaris* L.) Bioactive Components in the Food and Pharmaceutical Industries.” Critical Reviews in Food Science and Nutrition 64, no. 3: 708–739. 10.1080/10408398.2022.2108367. 35972148

[fsn370167-bib-0071] Tutunchi, P. , L. Roufegarinejad , H. Hamishehkar , and A. Alizadeh . 2019. “Extraction of Red Beet Extract With β‐Cyclodextrin‐Enhanced Ultrasound Assisted Extraction: A Strategy for Enhancing the Extraction Efficacy of Bioactive Compounds and Their Stability in Food Models.” Food Chemistry 297: 124994. 10.1016/j.foodchem.2019.124994. 31253277

[fsn370167-bib-0072] Wichchukit, S. , and M. O'Mahony . 2015. “The 9‐Point Hedonic Scale and Hedonic Ranking in Food Science: Some Reappraisals and Alternatives.” Journal of the Science of Food and Agriculture 95, no. 11: 2167–2178. 10.1002/jsfa.6993. 25378223

[fsn370167-bib-0073] Zin, M. M. , E. Márki , and S. Bánvölgyi . 2020. “Conventional Extraction of Betalain Compounds From Beetroot Peels With Aqueous Ethanol Solvent.” Acta Alimentaria 49, no. 2: 163–169. 10.1556/066.2020.49.2.5.

